# Spirituality, religiosity and the mental health consequences of social isolation during Covid-19 pandemic

**DOI:** 10.1177/0020764020970996

**Published:** 2021-09

**Authors:** Giancarlo Lucchetti, Leonardo Garcia Góes, Stefani Garbulio Amaral, Gabriela Terzian Ganadjian, Isabelle Andrade, Paulo Othávio de Araújo Almeida, Victor Mendes do Carmo, Maria Elisa Gonzalez Manso

**Affiliations:** 1 School of Medicine, Federal University of Juiz de Fora, Juiz de Fora, Brazil; 2 School of Medicine, São Camilo University Center, São Paulo, SP, Brazil; 3 School of Medicine, Federal University of Mato Grosso, Cuiabá, Brazil

**Keywords:** COVID-19, pandemic, spirituality, religion and medicine, mental health

## Abstract

**Background:**

Evidence shows that religiosity and spirituality (R/S) are highly used in critical moments of life and that these beliefs are associated with clinical outcomes. However, further studies are needed to assess these beliefs during the COVID-19 pandemic.

**Aims:**

To evaluate the use of R/S during the COVID-19 pandemic in Brazil and to investigate the association between R/S and the mental health consequences of social isolation.

**Methods:**

Cross-sectional study conducted in May 2020. Online surveys were carried out assessing sociodemographics, R/S measures, and social isolation characteristics and mental health consequences (hopefulness, fear, worrying and sadness). Adjusted regression models were used.

**Results:**

A total of 485 participants were included from all regions of Brazil. There was a high use of religious and spiritual beliefs during the pandemic and this use was associated with better mental health outcomes. Lower levels of worrying were associated with greater private religious activities (OR = 0.466, CI 95%: 0.307–0.706), religious attendance (OR = 0.587, CI 95%: 0.395–0.871), spiritual growth (OR = 0.667, CI 95%: 0.448–0.993) and with an increase in religious activities (OR = 0.660, CI 95%: 0.442–0.986); lower levels of fear were associated with greater private religious activities (OR = 0.632, CI 95%: 0.422–0.949) and spiritual growth (OR = 0.588, CI 95%: 0.392–0.882) and, lower levels of sadness (OR = 0.646, CI 95%: 0.418–0.997) were associated with spiritual growth. Finally, hope was associated with all R/S variables in different degrees (ranging from OR = 1.706 to 3.615).

**Conclusions:**

R/S seem to have an important role on the relief of suffering, having an influence on health outcomes and minimizing the consequences of social isolation. These results highlight the importance of public health measures that ensure the continuity of R/S activities during the pandemic and the training of healthcare professionals to address these issues.

## Introduction

Since the end of January 2020, the World Health Organization (WHO) has officially declared the COVID-19 epidemic as a public health emergency of international interest (Lake, 2020). In this context, the experience reported in previous humanity crises have shown that individuals exposed to these situations can usually develop a series of mental health problems, such as post-traumatic stress disorder, depression, generalized anxiety, panic, phobias and substance abuse (Acierno et al., 2007; Mason et al., 2010).

The COVID-19 pandemic is having and will have important consequences, not only to the physical health, but also to the mental health of the population (Pfefferbaum & North, 2020). A recent systematic review (Rogers et al., 2020) have identified that these psychiatric consequences can have implications for both the short and long terms and will probably increase the prevalence of depression, anxiety, fatigue, insomnia and post-traumatic stress.

Currently, social distancing is considered an effective measure to reduce the high infectivity of the disease, so most countries of the world have adopted this procedure in order to minimize contagion and consequences for public health systems (Usher et al., 2020). If on the one hand, social distancing has reduced the pressure on hospital beds; on the other hand, it has caused other consequences such as unemployment, lack of medical appointments and impairment in mental health (Rogers et al., 2020; VanderWeele, 2020b).

The exposure to the pandemic stress and the social isolation seem to increase mental health problems, creating challenges for public health managers and mental healthcare professionals (Banerjee, 2020). In this situational framework, individuals become more vulnerable to negative feelings (e.g. anxiety and fear) and irrational ideas, which may be intensified by news reports on the increase in cases and deaths (Lima et al., 2020).

There are several strategies to reduce these negative consequences of social isolation, such as using social medias, contacts with family and friends through digital technologies, online classes, virtual workplaces and the use of religious and spiritual beliefs (Galea et al., 2020; Koenig, 2020). In fact, religiosity and spirituality (R/S) have been used to handle crisis situations and stress for a long time (Ebadi et al., 2009; Schuster et al., 2001; Thune-Boyle et al., 2006). Studies have already reported the effects of R/S in physical and mental health, promoting higher levels of life satisfaction, well-being, sense of purpose, meaning of life, hope, optimism and lower rates of anxiety, depression and substance abuse (Koenig, 2012).

The previous evidence suggests that R/S could be an important tool for the population to deal with the new pandemic reality. However, despite the fact that several authors have already proposed R/S interventions during the pandemic (Del Castillo et al., 2020; Ferrell et al., 2020), few studies (Pirutinsky et al., 2020; Weinberger-Litman et al., 2020) have investigated how these beliefs are used and whether they can minimize the social isolation consequences during this pandemic.

In an attempt to bridge this gap, the present study aims to evaluate the use of religious and spiritual beliefs during the COVID-19 pandemic in Brazil and to investigate the association between R/S and the mental health consequences of the social isolation (i.e. hopefulness, fear, worrying and sadness).

## Methods

This was an observational cross-sectional quantitative study carried out in the month of May 2020 (the peak of the COVID-19 pandemic in Brazil). This study was submitted and approved by the Ethics Research Committee of the São Camilo University Center (São Paulo, Brazil) under approval number 4.047.769, CAAE 31154920.0.0000.0062 and all participants consent to participate signing an online form.

### Eligibility criteria

In order to be included participants should: have at least 18 years old, live in Brazil (all regions were accepted), be experiencing the pandemic of COVID-19, be able to read and write in Portuguese and be in social isolation for at least 10 days (this cutoff was used to guarantee that participants were facing the consequences of social isolation).

### Procedures

Participants were invited online by the researchers using social medias and emails. This was carried out using a ‘snowball technique’ (i.e. nonprobability sampling where study subjects recruit future subjects from among their contacts) in a sense that the researchers used their social medias and emails and asked their own contacts to disseminate the study to the largest number of individuals. The questionnaire was available for answers during a period of four complete weekdays.

### Instrument

The questionnaire took approximately 10 minutes to be filled in and it was delivered online using Google Forms^®^. The first page of the form was an online consent and the participant had the opportunity to agree or not with the terms of the study. The following questions were used:

a) Sociodemographic data: age, gender, profession, marital status and education.
b) Religious and Spiritual Beliefs:
  - Religious affiliation: respondent could choose different religious affiliations (e.g. Catholics, Spiritists, Evangelicals) or could report not having a religion. Those not having a religion were separated in spiritual but not religious or non-believers (e.g. atheists).
  - Religious attendance: the question ‘How often do you attend church or other religious meetings?’ based on the first item of the Duke Religion Index validated into Portuguese was used (Lucchetti, Lucchetti, Peres, Leão, et al., 2012). Answers were adapted from the original version using a likert of eight items ranging from ‘Never’ to ‘Everyday’.
  - Religious private activities: the question ‘How often do you spend time in private religious activities, such as prayer, meditation or Bible study?’ based on the first item of the Duke Religion Index validated into Portuguese was used (Lucchetti, Lucchetti, Peres, Leão, et al., 2012). Answers were adapted in a likert of eight items ranging from ‘Never’ to ‘Everyday’.
  - Importance of Religion: a self-reported religiousness question from the Duke NIMH Epidemiologic Catchment Area Study in 1994 and adapted to the Brazilian context was used (Lucchetti, Lucchetti, Peres, Moreira-Almeida, & Koenig, 2012), asking about the importance of religion to life. Five point Likert answers ranged from ‘Not at all’ to ‘Very important’
  - Religion/Spirituality helping in the social isolation: a question asking how R/S was helping participants in coping with the social isolation was created by authors with five possible answers: ‘not helping’ to ‘Helping a lot’.
  - Spiritual growth: participants were asked whether the pandemic was responsible to promote a spiritual growth. This question was developed by researchers and was contextualized to the pandemic moment. Possible answers range in 5 points: ‘Not growing at all’ to ‘Growing a lot’.
  - Religious activities during social isolation: another question created by researchers aiming to understand if the religious practices were impacted by the social isolation. Three possible answers were allowed: ‘Improved’, ‘No change’ and ‘Worsened’.
c) Social isolation characteristics: questions concerning the social isolation were developed, including the Region of social isolation, the number of days in social isolation, if the participant was able to maintain his/her job or study at home, if the participant was having an income during social isolation and how many persons the participant was isolated with.
d) Social isolation consequences: after a discussion with researchers and based on the literature, some consequences of the social isolation were raised in order to create the questionnaire. Thus, fear, worrying, sadness and hopefulness were included as important markers of social isolation. Questions were developed as follows: ‘How afraid are you with the pandemic?’, ‘How worried are you with the pandemic?’, ‘How sad are you with the pandemic?’ and ‘How hopeful are you with the pandemic?’. The possible answers for each question were divided into four categories: ‘Very’, ‘Little’, ‘Not al all’ and ‘Indifferent’.

### Statistical analysis

Descriptive analyses were carried for the sociodemographic data, spiritual/religious beliefs, social isolation characteristics and its mental health consequences using mean and standard deviation for continuous measures and absolute numbers and percentages for ordinal or categorical measures. After this descriptive analysis, dichotomous variables were created for the Likert items.

In the inferential analyses, logistic regression models were conducted using dichotomous religious and spiritual measures (e.g. Religious attendance: 1 = Once a week or more and 0 = Less than once a week) as independent variables and the mental health consequences of social isolation (e.g. How afraid are you with the pandemic? 1 = Very afraid and 0 = Little/Not al all/Indifferent) as dependent variables. Models were adjusted for sociodemographics (Model 1: age, gender, profession, Marital Status, Education) and for the social isolation characteristics (Model 2: Region, number of days in social isolation, able to maintain your job or study at home, still having an income during social isolation, isolated with how many persons). Odds ratio were reported with 95% Confidence intervals.

Analyses were carried out in SPSS 20.0 (SPSS Inc.) and a *p*-value ⩽ 0.05 was considered significant.

## Results

From a total of 544 responses in the Google Forms, 485 participants were included in the final analysis. Reasons for exclusion were: participant didn’t agree with the consent term (*n* = 1), missing information (*n* = 2), less than 10 days of social isolation (*n* = 30) and duplicate data (*n* = 26).

The sample was mostly composed by females (79.2%), students (54.2%), with complete or incomplete university level (86.6%), single (70.3%), with mean age of 31.8 (SD 13.7) years and from all regions of Brazil. Concerning the religious and spiritual characteristics, 75.7% had a religious affiliation (42.8% of them Catholics), 17.5% did not have an affiliation but considered themselves ‘spiritual’ and 6.6% did not have a religious affiliation (Atheists and non believers). At least 44.1% attended to religious services once a week or more, 62.3% carried out religious private activities more than once a week and 76.7% considered religion important to their lives. Most participants (73.4%) reported that R/S was helping them to cope with the social isolation, 57.3% have experienced a spiritual growth and 33.8% believed that the social isolation has improved their religious activities (Table 1).

**Table 1.** Sociodemographic and spiritual/religious characteristics of the sample.

	*n*	%
Gender		
Female	384	79.2
Male	100	20.6
Did not declare	1	0.2
Education		
Incomplete or complete university level	420	86.6
Others	65	13.4
Marital status		
Single	341	70.3
Others	144	29.7
Region		
South/Southeast	301	62.1
North/Northeast/Central-West	184	37.9
Profession		
Student (University/College/High-School)	263	54.2
Health professional	70	14.4
Others	139	28.6
Retired	13	2.8
Religion		
None and not believe	32	6.6
None but spiritual	85	17.5
Religious	368	75.7
Catholics	157	42.8
Evangelicals	73	19.8
Spiritists	98	26.6
Others	40	10.8
Religious attendance		
Once a week or more	214	44.1
Less than once a week	271	55.9
Frequency of religious private activities		
More than once a week	302	62.3
Once a week or less	183	37.7
Importance of religion to his/her life		
Important or very important	372	76.7
Moderately, little or not important	113	23.3
Religion/Spirituality helping in the social isolation		
Helping or helping a lot	356	73.4
Moderately, little or not helping	129	26.6
Spiritual growth during social isolation		
Growing or growing a lot	278	57.3
Moderately, little or not growing	207	42.7
Social isolation has changed the religious activities		
Improved	164	33.8
No change or worsen	321	66.2
	Mean	*SD*
Age	31.89	13.79

Table 2 presents the characteristics and consequences of social isolation. The number of days in social isolation was 41.9 (SD 8.4) and the number of persons isolated together in the same household was 2.5 (SD 1.3). Most participants were able to maintain their routine of job or study (84.1%) and their income (50.5%). As a consequence of social isolation, 31.5% of participants were ‘very afraid’, 65.2% were ‘very worried’ and 24.7% were ‘very sad’ with the pandemic. Despite these numbers, 60.4% reported being ‘very hopeful’ with this situation.

**Table 2.** Social Isolation characteristics and consequences.

	*n*	%
Were you able to maintain your job or study at home?		
Yes	408	84.1
No	77	15.9
Are you still having an income during social isolation?		
I have never had an income	170	35.1
No	70	14.4
Yes	245	50.5
How afraid are you with the pandemic?		
Very afraid	153	31.5
Little/Not at all/Indifferent	332	68.5
How worried are you with the pandemic?		
Very worried	316	65.2
Little/Not at all/Indifferent	169	34.8
How sad are you with the pandemic?		
Very sad	120	75.3
Little/Not at all/Indifferent	365	24.7
How hopeful are you with the pandemic?		
Very hopeful	293	60.4
Little/Not at all/Indifferent	192	39.6
	Mean	*SD*
Number of days in social isolation	41.92	8.49
Persons isolated together in the household	2.56	1.34

The logistic regression models for the association between religious variables and mental health outcomes are shown in Table 3. After adjustment: (a) participating in private religious activities was associated with lower levels of fear (OR = 0.632, CI 95%: 0.422–0.949, *p* < 0.05) and lower levels of worrying (OR = 0.466, CI 95%: 0.307–0.706, p < 0.001), (b) Higher levels of spiritual growth was associated with lower levels of fear (OR = 0.588, CI 95%: 0.392–0.882, *p* < 0.05), lower levels of worrying (OR = 0.667, CI 95%: 0.448–0.993, *p* < 0.05) and lower levels of sadness (OR = 0.646, CI 95%: 0.418–0.997, *p* < 0.01), (c) higher levels of religious attendance was associated with lower levels of worrying (OR = 0.587, CI95%: 0.395–0.871, *p* < 0.01), (d) improving religious activities was associated with lower levels of worrying (OR = 0.660, CI 95%: 0.442–0.986) and (e) all religious variables were associated with hoping in different degrees (ranging from OR = 1.706 to 3.615).

**Table 3.** Adjusted Logistic Regression models for the association between religious variables and social isolation outcomes.

	Unadjusted	Model 1	Model 2
How afraid are you with the pandemic? 1 = Very afraid 0 = Little/Not at all/Indifferent			
Religious attendance (Once a week or more)	0.807 (0.547–1.190)^NS^	0.717 (0.479–1.074)^NS^	0.718 (0.477–1.080)^NS^
Religious private activities (More than once a week)	0.717 (0.485–1.061)^NS^	0.648 (0.434–0.969)*	0.632 (0.422–0.949)*
Social isolation has changed the religious activities (Improved)	1.249 (0.837–1.863)^NS^	1.176 (0.782–1.768)^NS^	1.156 (0.767–1.744)^NS^
Importance of religion (Important or Very Important)	1.448 (0.902–2.326)^NS^	1.361 (0.836–2.214)^NS^	1.374 (0.841–2.246)^NS^
Religion/Spirituality helping in the isolation (Helping/Helping a lot)	0.813 (0.530–1.246)^NS^	0.701 (0.448–1.097)^NS^	0.710 (0.452–1.115)^NS^
Spiritual growth during social isolation (Improved)	0.686 (0.467–1.009)^NS^	0.597 (0.399–0.894)*	0.588 (0.392–0.882)*
How worried are you with the pandemic? 1 = Very worried 0 = Little/Not at all/Indifferent			
Religious attendance (Once a week or more)	0.657 (0.451–0.957)*	0.616 (0.418–0.909)*	0.587 (0.395–0.871)**
Religious private activities (More than once a week)	0.510 (0.341–0.763)***	0.476 (0.315–0.719)***	0.466 (0.307–0.706)***
Social Isolation has changed the religious activities (Improved)	0.674 (0.457–0.996)*	0.651 (0.437–0.969)*	0.660 (0.442–0.986)*
Importance of religion (Important or Very Important)	1.086 (0.700–1.684)^NS^	1.026 (0.655–1.609)^NS^	1.030 (0.654–1.622)^NS^
Religion/Spirituality helping in the isolation (Helping/Helping a lot)	0.830 (0.540–1.275)^NS^	0.765 (0.491–1.192)^NS^	0.773 (0.494–1.209)^NS^
Spiritual growth during social isolation (Improved)	0.737 (0.503–1.080)^NS^	0.681 (0.459–1.010)^NS^	0.667 (0.448–0.993)*
How sad are you with the pandemic? 1 = Very sad 0 = Little/Not at all/Indifferent			
Religious attendance (Once a week or more)	0.763 (0.501–1.162)^NS^	0.689 (0.446–1.065)^NS^	0.695 (0.447–1.081)^NS^
Religious private activities (More than once a week)	0.922 (0.604–1.409)^NS^	0.867 (0.563–1.335)^NS^	0.837 (0.540–1.295)^NS^
Social Isolation has changed the religious activities (Improved)	1.431 (0.934–2.192)^NS^	1.365 (0.885–2.104)^NS^	1.423 (0.916–2.210)^NS^
Importance of religion (Important or Very Important)	1.689 (0.938–2.691)^NS^	1.519 (0.886–2.602)^NS^	1.474 (0.854–2.542)^NS^
Religion/Spirituality helping in the isolation (Helping/Helping a lot)	0.941 (0.592–1.496)^NS^	0.863 (0.533–1.398)^NS^	0.889 (0.546–1.447)^NS^
Spiritual growth during social isolation (Improved)	0.737 (0.487–1.116)^NS^	0.669 (0.435–1.028)^NS^	0.646 (0.418–0.997)*
How hopeful are you with the pandemic? 1 = Very hopeful 0 = Little/Not at all/Indifferent			
Religious attendance (Once a week or more)	2.177 (1.491–3.179)***	2.093 (1.407–3.112)***	2.208 (1.473–3.309)***
Religious private activities (More than once a week)	3.081 (2.101–4.518)***	2.930 (1.969–4.361)***	3.064 (2.047–4.587)***
Social isolation has changed the religious activities (Improved)	1.600 (1.078–2.374)*	1.723 (1.141–2.603)*	1.706 (1.126–2.585)*
Importance of religion (Important or Very Important)	2.373 (1.547–3.642)***	2.008 (1.287–3.131)**	2.096 (1.336–3.290)***
Religion/Spirituality helping in the isolation (Helping/Helping a lot)	4.089 (2.673–6.254)***	3.491 (2.245–5.427)***	3.615 (2.309–5.658)***
Spiritual growth during social isolation (Improved)	3.797 (2.587–5.574)***	3.458 (2.320–5.155)***	3.615 (2.413–5.416)***

Model 1: Age, gender, profession, Marital Status, Education; Model 2: Region, number of days in social isolation, able to maintain your job or study at home, still having an income during social isolation, isolated with how many persons. NS = not significant.

* *p* < .05. **p ⩽ .01. ***p ⩽ .001.

## Discussion

The present study investigated the association between R/S and the mental health consequences of social isolation during the COVID-19 pandemic in Brazil. Our findings indicated that there was a high use of religious and spiritual beliefs during the pandemic and that this use was associated with better health outcomes, as evidenced by the higher levels of hopefulness and lower levels of fear, worrying and sadness in the more religious and spiritual participants. These results highlight the role of R/S in coping with adverse situations and will be discussed below.

In fact, R/S have been proposed as important tools used to face suffering caused by trauma and stress based on specific strategies also known as spiritual and religious coping (Harrison et al., 2001). Coping can be used by individuals in a positive (e.g. meaning to be found, spiritual connection and benevolent religious reappraisals) or a negative way (e.g. religious struggle, punishment and reappraisal of God’s power) and, for this reason, it could have positive or deleterious consequences (Pargament et al., 1998). Several studies (Ebadi et al., 2009; Schuster et al., 2001; Thune-Boyle et al., 2006) have already shown that coping is frequently used in moments of stress. A previous study has shown that 90% of participants from a representative US sample (Schuster et al., 2001) reported having turned to religion to deal with the terrorist attacks on September 11, 2001. Similar results were also observed in war scenarios (Ebadi et al., 2009) and in chronic diseases (Thune-Boyle et al., 2006).

In the specific case of the COVID-19 pandemic, our results seem to be confirmed, since three out of four participants in this research declare S/R was helping them to cope with the social isolation and more than half have experienced a spiritual growth. Although there are few studies available about the current pandemic, Google searches about prayer doubled for every 80,000 new cases of COVID-19 (Taylor, 2020), besides a great demand for religious and spiritual hotlines (Ribeiro et al., 2020) and religious community conference calls (Galiatsatos et al., 2020) were observed.

Not only the search for R/S seems to have increase, but also these beliefs seem to have a remarkable influence in mental health during the pandemic. Our findings revealed that R/S were associated with better health outcomes such as lower levels of sadness, fear and worrying, while they were also associated with higher levels of hope. Although such results have been extensively discussed in the previous decades (Koenig, 2012), few studies have assessed the actual pandemic moment.

One of the few studies that investigated the influence of R/S on health outcomes during the COVID-19 pandemic (Weinberger-Litman et al., 2020) included 303 members of North American religious communities and found no association between religious commitment with distress or anxiety. According to the authors, the lack of association is because this population is very religious and composed of members of specific religious traditions which may lead to a low variability of responses and minimized the statistical power. In the present study, this problem was reduced since our sample was more heterogeneous.

Another study (Pirutinsky et al., 2020) has investigated 419 American Orthodox Jews and found that positive religious coping, intrinsic religiosity and trust in God strongly correlated with less stress and more positive impact, while negative religious coping and mistrust in God correlated with the inverse. These results are similar to our findings indicating that faith may promote resilience during crisis.

Among various R/S variables assessed in our study, it is possible to note that spiritual growth and private religious activities were the most associated with positive outcomes during social isolation. These results are interesting because there is a tendency in previous studies to present religious attendance as the most important variable (VanderWeele, 2020a). In the COVID-19 pandemic, the restriction of mobility and social contact prevents routine religious attendance (Nicola et al., 2020) and, for this reason, other R/S strategies seem to offer the same benefits found by organizational religiosity.

According to Koenig (Koenig, 2020), social distance is an opportunity to develop a stronger relationship with God by personal religious activities, reading Holy scriptures and listening to or watching inspiring radio, podcast or TV programs. In the present study, it was possible to verify that at least one third of the participants reported having increased their religious activities during the pandemic. Several other initiatives have also been proposed to reduce the problem of the closure of religious services, such as prayer meetings on the balconies of buildings (Frei-Landau, 2020), religious celebrations in a place where people gather without leaving their vehicles (Modell & Kardia, 2020) and spiritual hotlines (Ribeiro et al., 2020).

Inside the hospitals, where feelings of brevity, finitude, vulnerability and impotence are more evident (Hart, 2020), the spiritual care of the health team, chaplains and local religious groups gains importance (Ferrell et al., 2020; Taylor, 2020). This holistic care can be provided by using compassionate presence, listening and communication skills (Puchalski et al., 2020), openness of the healthcare professional to the patient’s beliefs (Taylor, 2020) and also using telecommunication devices (e.g. telephone-based chaplaincy) for patients in isolation (Sprik et al., 2020). In addition to the genuine attention of the healthcare team, webmeetings with the loved ones and religious leaders seems to reduce loneliness and stress (Ferrell et al., 2020). Finally, R/S may help assisting the bereavement and suffering of the health team and families (Puchalski et al., 2020).

The present study has some limitations that should be considered while interpreting our results. First, this is a cross-sectional study and cause-effect relationships are not allowed. Second, our sample was mostly composed of women with high education levels. This is probably a result of the use of ‘snow ball’ sampling procedure which may have imbalanced the sample. Women are usually more religious than men and this could have impacted the high levels of R/S evidenced in our study. More Brazilian representative studies are needed. Third, Brazil is a highly religious country and more studies are needed in secular societies. Forth, single question measures were used to assess sadness, hope, worrying and fear. More complex instruments may be used in future studies. Finally, as reported previously, coping strategies can have negative and positive influences on mental health. However, our questionnaire was not able to identify the negative aspects of coping, which could have an impact in the outcomes.

## Conclusion

Our findings corroborate with the opinion of previous authors that R/S seems to have an important role in reducing suffering, influencing health outcomes and minimizing the consequences of social distance (Hart, 2020; Hart & Koenig, 2020; Koenig, 2020). This data confirms the importance of public health measures to ensure the continuity of religious and spiritual activities during the pandemic and the training of healthcare professionals to address spiritual and religious beliefs of patients and families. Healthcare professionals must be aware of the positive and negative use of individuals’ beliefs in order to provide the most comprehensive care possible.
